# Can the French version of the short Örebro Musculoskeletal Pain Screening Questionnaire or its subsets predict the evolution of patients with acute, (sub) acute and chronic pain?

**DOI:** 10.1186/s12891-021-04944-9

**Published:** 2022-04-01

**Authors:** Natalya Korogod, Arnaud Steyaert, Olivier Nonclercq, Emmanuelle Opsommer, Anne Berquin

**Affiliations:** 1 School of Health Sciences (HESAV), University of Applied Sciences and Arts Western Switzerland (HES-SO), Avenue de Beaumont, 21, 1011 Lausanne, Switzerland; 2 Department of Anesthesiology, Cliniques universitaires Saint-Luc, Avenue Hippocrate 10/1650, 1200 Brussels, Belgium; 3 Institute of Neuroscience, Université Catholique de Louvain, Brussels, Belgium; 4 Grand Hôpital de Charleroi, Service de Médecine Physique et Réadaptation, 6061 Montignies-sur-Sambre, Belgium; 5 Department of Physical and Rehabilitation Medicine, Cliniques universitaires Saint-Luc, Avenue Hippocrate 10/1650, 1200 Brussels, Belgium

**Keywords:** Low back pain, Secondary prevention, Örebro Musculoskeletal Pain Screening Questionnaire, Psychosocial, Screening

## Abstract

**Background:**

Prevention of chronic pain relies on accurate detection of at-risk patients. Screening tools have been validated mainly in (sub) acute spinal pain and the need of more generic tools is high. We assessed the validity of the French version of the short Örebro Musculoskeletal Pain Screening Questionnaire (ÖMPSQ) in patients with a large range of pain duration and localization.

**Methods:**

First, we re-analyzed data from a 6-month longitudinal study of 73 patients with (sub) acute spinal pain consulting in secondary line settings. Secondly, we performed a new 12-month longitudinal study of 542 primary care patients with (sub) acute and chronic pain in different localizations (spinal, limbs, “non-musculoskeletal”). The area under the receiver operating characteristic curve and cutoff scores were computed and compared for different subpopulations and ÖMPSQ subscores.

**Results:**

Data from patients suffering from (sub) acute and chronic spinal pain consulting in both primary and secondary care settings confirmed the validity of the short French ÖMPSQ version and its subsets. In the primary care cohort, the performance of the questionnaire and its psychosocial subscore was variable but at least “fair” in most populations ((sub) acute and chronic, spinal and limb pain). Cutoff scores showed quite large variability depending on the outcome and the subpopulation considered.

**Conclusions:**

These results confirm the usefulness of the short French ÖMPSQ for prediction of the evolution of (sub) acute and chronic patients with spinal and limb pain, whatever its duration. However, increasing population heterogeneity results in slightly worse predictive performance and largely variable cutoff scores. Consequently, it might be difficult to choose universal cutoff scores and other criteria, such as patients’ values and the available resources for patient management, should be taken into account.

**Supplementary Information:**

The online version contains supplementary material available at 10.1186/s12891-021-04944-9.

## Background

Chronic pain is one of the main public health burdens. It’s high costs – ranging from five to €32 billion worldwide per year for low back pain (LBP) [1, 2] – justify the interest of prevention strategies. As acute pain is common and usually resolves favorably, interventions addressing all pain patients would not be cost-effective and could even be counterproductive. Indeed, while approaches specifically targeting high-risk patients significantly reduce the long-term impact of acute low-back pain, over-treating low-risk patients might increase long-term disability [3]. These strategies rely on early identification of the 10-20% of the patients with (sub) acute pain that are at risk of developing chronic disabling pain (about 65% of LBP patients still report pain one year after pain onset [4], but only 10-20% are still off work [5]). Risk factors are known to be mainly psychosocial (“yellow flags”) and involve beliefs and behaviors such as catastrophizing, fear-avoidance and reduced activity level [6]. Screening tools such as the STarT Back Screening Tool (SBT) [7] or the Örebro Musculoskeletal Pain Screening Questionnaire (ÖMPSQ) [8, 9] have been developed for identification of at-risk LBP patients. Questions concerning these questionnaires relate to the potential interest of shorter versions as well as their validity in populations with different pain etiology and/or duration.

Several versions of the ÖMPSQ questionnaire have been validated (full 25-items [8], short 10- [9] and 2-items versions [8–10]). In this questionnaire, the total score determines risk groups. Recent systematic reviews [11, 12] showed that full and short ÖMPSQ versions had similar predictive power, ranging between “acceptable” and “excellent”, for functional limitations and return to work (RTW) outcomes. The ÖMPSQ has been validated in different languages [8, 10, 13–19]. For French-speaking countries, the full version has been validated for acute [13] and chronic LBP [20] and the short version for chronic LBP [10, 20]. While ÖMPSQ is used to identify risk groups, stratification of LBP patients for treatment is done with SBT, which has two scoring options (total and psychosocial) that allocate patients to three categories: high “psychosocial”, medium “physical” and low risks [7]. One could wonder if the distinction of a psychosocial subscore provided by the SBT could also be useful with the ÖMPSQ.

The most common etiologies of chronic pain are musculoskeletal disorders, among which spinal pain is the most frequent [21]. Currently available screening questionnaires focus only (SBT) or mainly (ÖMPSQ) on spinal pain [7–9]. The ÖMPSQ was also partly validated for musculoskeletal pain (MSKP) [11]. Other conditions such as orofacial or visceral pain also contribute to the contingent of chronic pain. Several systematic reviews suggested that risk factors are mostly comparable across pain conditions. For MSKP [22–24] and pelvic pain [25], prominent factors were pain intensity and duration, the number of pain sites, previous pain episodes, high disability, anxiety, depression, somatization, coping factors and possibly age and high body mass index. The involvement of anxiety and depression was also observed for headache [26], irritable bowel syndrome [27, 28] and post-surgical pain [29, 30]. The similarity of risk factors asks the question of a tool that could reasonably predict evolution across pain conditions. Some work suggested that this might be feasible: a statistical tool based on pain intensity, duration and interference as well as depression predicting the evolution of LBP was also valid for headache, orofacial pain [31] and MSKP (but with different cutoff scores) [32]. The ÖMPSQ addresses most of these factors and its wording does not specifically refer to LBP. It might thus be a good starting point in the search of a generic screening tool.

Finally, chronic pain is not a fixed state and should rather be viewed dynamically as “clinically significant pain likely to be present one or more years in the future” [33]. In this perspective, prognostic tools are interesting for patients suffering from pain lasting more than 3-6 months, to identify factors that could be addressed in a tertiary prevention strategy. The SBT and ÖMPSQ have been validated mainly with (sub) acute patients, even if some studies used a mixed cohort of (sub) acute and chronic patients [9, 34–37] and one cohort assessed chronic LBP patients [10, 20].

The aim of this paper is to further assess the validity and cutoff scores of the short French ÖMPSQ and its subsets in different situations: (1) primary and secondary care settings, (2) (sub) acute and chronic pain and (3) spinal and non-spinal (musculoskeletal and non-musculoskeletal) pain.

To this end, two patient cohorts were used. First, we re-analyzed data from previous work validating the French full ÖMPSQ [13], a 6-month longitudinal study of 73 patients with spinal pain consulting in secondary line settings. Then, a new 12-month longitudinal study of 542 primary care patients suffering from spinal, musculoskeletal or non-musculoskeletal pain was performed.

## Methods

### Re-analysis of a previous cohort of patients suffering from spinal pain consulting in a secondary line setting

We applied the approach of Linton et al. [9], where items were extracted and analyzed from the full ÖMPSQ version. For that purpose, we re-analyzed data from our previous study [13].

#### *Summary of the methods of the initial study* [13]

Study participants were a convenience sample of patients suffering from non-specific (sub) acute low back or neck pain, presenting at the emergency facility or the outpatient consultation of the Physical and Rehabilitation Medicine department at the Cliniques universitaires Saint-Luc (Brussels). Inclusion criteria were the presence of pain for less than three months and a cumulated sick leave due to pain of less than 6 months in the past year. Exclusion criteria were inability to read and understand French and the presence of “red flags” [38]. Participants completed the full French ÖMPSQ at the day of inclusion (t0) and 6 months later (t6).

#### Data Analysis and Statistics

Scoring for the short ÖMPSQ was performed according to previous studies [8, 9] by summing up the scores for individual items (Table 1), after inversion of items #3, #4, and #8. Scores may range from 1 to 100. A psychosocial subscore was computed from items #5 to #10 (range 0-60). Only ≤5% of missing items was accepted and the score was adjusted using the average response of a given patient as an estimate of missing values [8].

**Table 1:** French version of the short ÖMPSQ version^a^

	Item	Concept domain	Notation^**b**^
**1**	Depuis combien de temps avez-vous vos douleurs actuelles?	Douleur	1-10
**2**	Quelle était l’intensité de votre douleur durant les sept derniers jours?	Douleur	0-10
	Voici une liste de deux activités. Veuillez entourer le chiffre qui décrit le mieux votre capacité actuelle à participer à chacune de ces activités.		
**3**	Je peux faire un travail léger pendant une heure.	Perception de soi	0-10, notation inversée
**4**	Je peux dormir la nuit.	Perception de soi	0-10, notation inversée
**5**	Dans quelle mesure vous êtes-vous senti tendu ou anxieux au cours de la dernière semaine?	Détresse	0-10
**6**	À quel point avez-vous été gêné par un sentiment de dépression au cours de la dernière semaine?	Détresse	0-10
**7**	À votre avis, quelle est l’ampleur du risque que votre douleur actuelle devienne persistante?	Attente retour au travail	0-10
**8**	À votre avis, quelles sont les chances que vous soyez capable de travailler dans six mois?	Attente retour au travail	0-10, notation inversée
**9**	Une augmentation de la douleur indique que je devrais arrêter ce que je fais jusqu’à ce que la douleur diminue.	Croyance de peur et d’évitement	0-10
**10**	Je ne devrais pas faire mes activités normales, y compris mon travail, avec ma douleur actuelle.	Croyance de peur et d’évitement	0-10

*ÖMPSQ* Örebro Musculoskeletal Pain Screening Questionnaire

^a^Adapté de Linton et al. [9]

^b^Notes plus élevées indiquent un risque plus élevé de développer une incapacité liée à la douleur

Three outcome variables were derived from the ÖMPSQ [8, 9, 13]: pain (product of items #10 and #11 from the full questionnaire – pain intensity and frequency – range 0-100), function (sum of items #21 to #25 in the full questionnaire – ability to perform light work during an hour, to walk during an hour, to do usual housework, the weekly shopping, to sleep – range 0-50) and work absenteeism (item #6 – number of work days missed during the last 6 months – range 0-10). Cutoff scores for dichotomization of “recovered” versus “non-recovered” patients were ≤ 16 for the pain index, ≥ 45 for the function index, and ≤ 30 days of work absenteeism for the work outcome.

Correlations between the full and short ÖMPSQ versions were evaluated using the Kendall rank-order correlation test. Correlation coefficients of 0.10-0.29 represented a weak correlation, coefficients of 0.30-0.59 represented a moderate correlation and coefficients of 0.60 and above represented a strong correlation [39, 40]. The area under the ROC curve (AUC) was calculated for each ÖMPSQ version with the three outcome variables at the inclusion (t0) and at 6 months (t6) [13]. Threshold scores were calculated with the low cutoff score (distinguishing low and moderate risk) corresponding to a sensitivity of 80% and the high cutoff score (distinguishing moderate and high risk) corresponding to a specificity of 80%. ROC AUCs were interpreted according to the traditional academic point system: [0.90-1] = excellent; [0.80-0.89] = good; [0.70-0.79] = fair; [0.60-0.69] = poor; [0.50-0.59] = fail. The Chi-square test was used to verify the statistical significance of the difference between the ROC AUC [41, 42]. Correlations between the psychosocial subscore and the total score of the short ÖMPSQ were tested for each of the three outcomes by using linear regression, where R-squared (r^2^) indicated the strength of this relationship.

All analyses were performed using Microsoft Excel and IBM SPSS 23.0 software.

### New cohort of patients suffering from heterogeneous pain conditions followed in primary care

#### Procedure

This study was part of a large longitudinal study performed in general practice. Fifth-year medical students performing their one-month general practice internship (t0, Nov 2018) and their supervisors were offered to collaborate to this study. Detailed written instructions were provided and the investigators (AB and AS) were available for any questions. The study was approved by an independent ethical committee (2018/19JUI/258, Commission d’Éthique Biomédicale Hospitalo-Facultaire, Université Catholique de Louvain).

To avoid interference with the beginning of the working day, we instructed the students to ask the third scheduled patient of every day to take part in the study. If the patient refused, the student could ask the fourth, and so on. Patients received information on the purpose and procedures of the study. They filled in an informed consent form, a questionnaire comprising demographic and lifestyle information, pain presence and localization (Axis I of the IASP taxonomy of chronic pain syndromes [43]), short ÖMPSQ and their e-mail address if they agreed to be contacted later. Students encoded the data anonymously and sent it to one of the investigators (AS), together with the original paper questionnaires. Participating students received extra credits for their algology exam. Students who did not wish to participate received a similar number of credits upon completion of an alternative assignment. No gratification was offered to the patients or the supervisors.

One year later (t1, Nov 2019), the patients who agreed to be contacted again were sent an e-mail with a link to a secured online survey (Limesurvey [44]). The questionnaire was the same as at t0, with the addition of the EQ-5D-5L quality-of-life measure [45]. Up to two reminders were sent if the patients did not respond within 15 days.

#### Inclusion and exclusion criteria

Inclusion criteria were to be the third patient to come to the general practice consultation, whatever the reason for the consultation. If the patient declined participation, the fourth patient was offered to participate, and so on.

Exclusion criteria were age under 18, inability to answer questionnaires in French, and absence of a valid e-mail address.

#### Data analysis

Descriptive statistics were used to present participant demographic and clinical characteristics. In order to assess potential selection biases, we compared the demographic and clinical data of the patients who responded at t1 (group A) to those who did not (group B). Data from patients suffering from pain attributed to cancer were excluded from further analysis.

As participants did not complete the full 25-items ÖMPSQ, we could not use the same outcome variables as in the secondary care cohort. Several outcome variables were defined: presence of pain (“Do you currently suffer from pain, yes or no?”), items of the EQ-5D-5L and a composite “recovery score” computed as: [number of pain sites + pain intensity from item #2 of the short ÖMPSQ + 10-light work ability from item #3 of the short ÖMPSQ + 10-sleep quality from item #4 of the short ÖMPSQ]. This score could range from 0 (completely recovered) to 40 (maximal pain interference). For the ROC-analysis, outcome variables were dichotomized as follows: for the five dimensions of the EQ-5D-5L a score of 1–2 (no or slight problems) was considered good while scores of 3–5 (moderate to extreme problems) was considered as indicating bad health. The “recovery score” was considered as indicating good health if ≤ 12 (i.e., in average, not more than 3/10 in each of the items) or bad recovery if > 12.

ROC curves and their confidence intervals (estimated by bootstrapping) of the short ÖMPSQ and its subsets were calculated for all outcomes, except for the “Autonomy” item of the EQ-5D-5L, which showed a ceiling effect (96% patients had no difficulties or slight difficulties). We calculated ROC curves for the total cohort, as well as for different subgroups according to pain duration and localization. Patients were classified as suffering from (sub) acute pain if their pain lasted less than 3 months and from chronic pain if it lasted longer. We considered pain localized in the cervical or lumbosacral regions as *spinal pain*, pain localized in the scapular girdle, upper or lower limb as *limb pain* and other pain localization (head, thorax, abdomen, pelvic girdle, anus, perineal and genital area) as *probably non-MSKP*. Data from patients with pain at more than one site were excluded to the analysis made according to pain localization (but not to the analysis according to pain duration).

Statistical analysis was performed with Microsoft Excel and SAS JMP Pro 14.

## Results

### Demographic data

#### Secondary care cohort

Out of 91 patients who entered the study at t0, 73 (80%) sent completed questionnaires at t6. Demographics were similar between dropout patients and those who completed the study. Patients who completed the study presented the following characteristics: mean age 42.2 ± 10.7 years, 56% females, 86% native French speaking, 88% had paid jobs and 15 years of education after age 6. Most patients had one painful site, 45% reported pain on 2 to 5 sites, 64% had pain for < 6 weeks, 30% had pain lasting from 6-11 weeks. Eighty per cent had less than 30 days off work. More details can be found elsewhere [13].

#### Primary care cohort

**Fig. 1:**
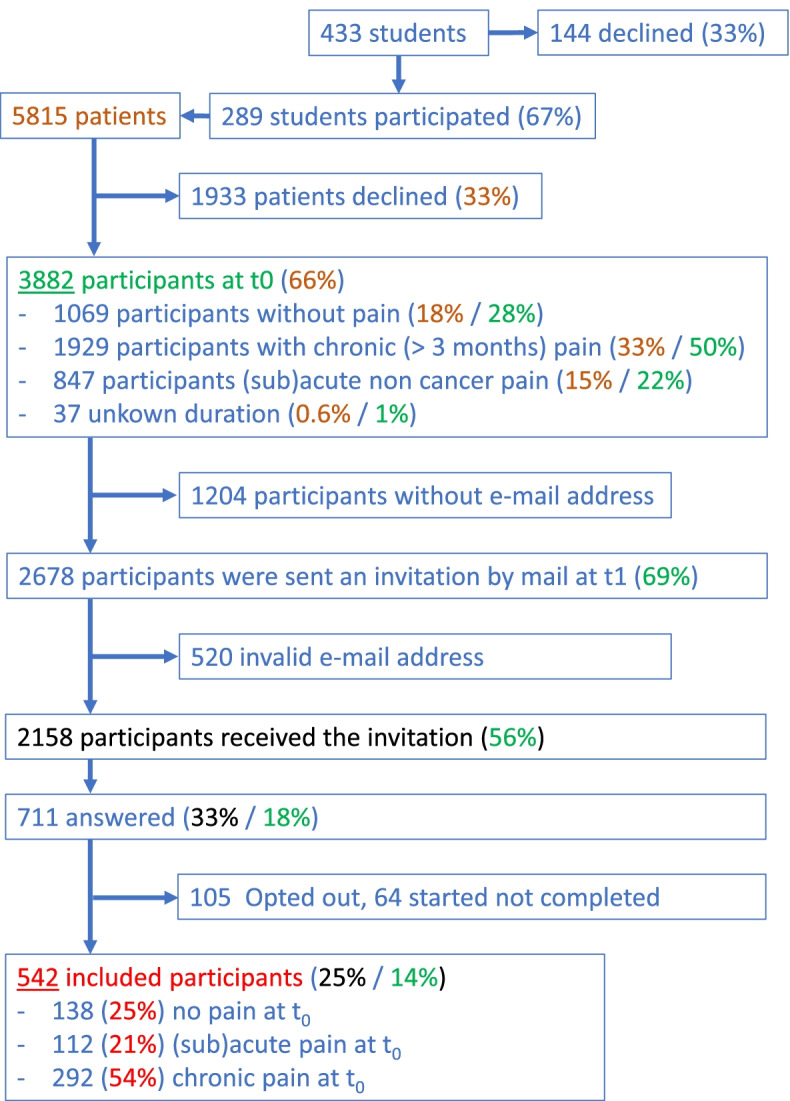
Flow chart of the primary care cohort study

Out of 433 medical students, 289 agreed to take part to the study. They approached 5815 patients, of which 3882 participated at t0 and 542 were included at t1 (see the study flowchart in Fig. 1).

Table 2 shows the demographic and clinical characteristics of the primary care cohort. The patients who replied to the online survey (group A) were slightly different from those who did not (group B): patients in the group A were younger and more often involved in a paid job. However, the distribution of pain duration and localization was not significantly different between groups. In the group A, most patients had more than one pain site, mainly spinal and limb pain. Among patients describing only one pain site, 48% had cervical or LBP, 39% had limb pain and 13% had “probably non-MSKP”. Within group A, females were more represented in pain than in no pain patients, patients with (sub) acute pain were more often involved in a paid job than other patients, patients with chronic pain were older, had a higher median number of pain sites and reported more spinal and limb pain than other patients.

**Table 2:** Demographic characteristics of patients from the primary care cohort

	Group A Patients having completed the study				p (groups A1 *vs* A2 *vs* A3)	Group B Drop-out patients	p (group A *vs* group B)
	A	A1	A2	A3			
	All patients	No pain at t0	Acute & subacute pain at t0	Chronic pain at t0			
n	542	138	112	292		3340	
Mean age (SD)	50.6 (15.0)	48.0 (17.6)	47.8 (15.5)	52.8 (13.2)	A1 *vs* A2: N.S. A1 *vs* A3: 0.002 A2 *vs* A3: 0.003	52.3 (18.6)	0.04
n Females (%)	337 (62%)	73 (53%)	80 (72%)	184 (63%)	All group: 0.007 A2 *vs* A3: N.S.	1961 (59%)	N.S.
Mean body mass index (SD)	26.3 (5.6)	25.1 (5.3)	26.2 (6.2)	26.9 (5.4)	A1 *vs* A2: N.S. A1 *vs* A3: 0.002 A2 *vs* A3: N.S.	26.4 (5.4)	N.S.
n paid job (%)	321 (60%)	81 (59%)	76 (69%)	164 (57%)	All groups: 0.0008 A2 *vs* A3: 0.0109	1462 (44%)	<0.0001
n retired (%)	137 (25%)	40 (29%)	16 (14%)	81 (28%)		1136 (34%)	
n no pain (%)	138 (25%)					931 (28%)	N.S.
n (sub) acute pain (%)	112 (21%)					735 (22%)	
n chronic pain (%)	292 (54%)					1637 (49%)	
Median number of pain sites	1	0	1	2	All groups: <0.0001 A2 *vs* A3: <0.0001	1	N.S.
n spinal pain (%)	281 (52%)	0	67 (60%)	214 (74%)	All groups: <0.0001 A2 *vs* A3: 0.0096	1575 (47%)	0.04
n limb pain (%)	261 (48%)	0	57 (51%)	204 (70%)	All groups: <0.0001 A2 *vs* A3: 0.0004	1479 (44%)	N.S.
n other pain sites (%)	98 (18%)	0	30 (27%)	68 (23%)	All groups: <0.0001 A2 *vs* A3: N.S.	641 (19%)	N.S.
n spinal and limb pain (%)	371 (69%)	0	96 (86%)	275 (95%)	All groups: <0.0001 A2 *vs* A3: 0.0070	2152 (64%)	N.S.
n “non MSK” pain only (%)	24 (4%)	0	11 (10%)	13 (4%)	All groups: <0.0001 A2 *vs* A3: 0.0503	208 (6%)	N.S.

*SD* Standard deviation

*N.S* not significant

Spinal pain: neck and/or low back

Limb pain: shoulder and arm(s) and/or lower limb(s)

Other pain sites: thorax and/or abdomen and/or pelvis and/or anus, perineum, genitals

“Non MSK” pain: patients suffering only of pain from other pain sites than spinal and limbs

### Predictive value of the short French ÖMPSQ and its subsets in patients suffering from spinal pain consulting in primary and secondary care settings

#### Comparison of the full and the short ÖMPSQ questionnaires

The re-analysis of data from our previous secondary care cohort showed a high correlation between the total scores of the full ÖMPSQ questionnaire and its short version, as well as different subscores (Kendall *r* = 0.781, *p*<0.01 at inclusion; r = 0.771, *p*<0.01 after 6 months).

#### Receiver operating characteristics of the short French ÖMPSQ and its subsets

**Fig. 2:**
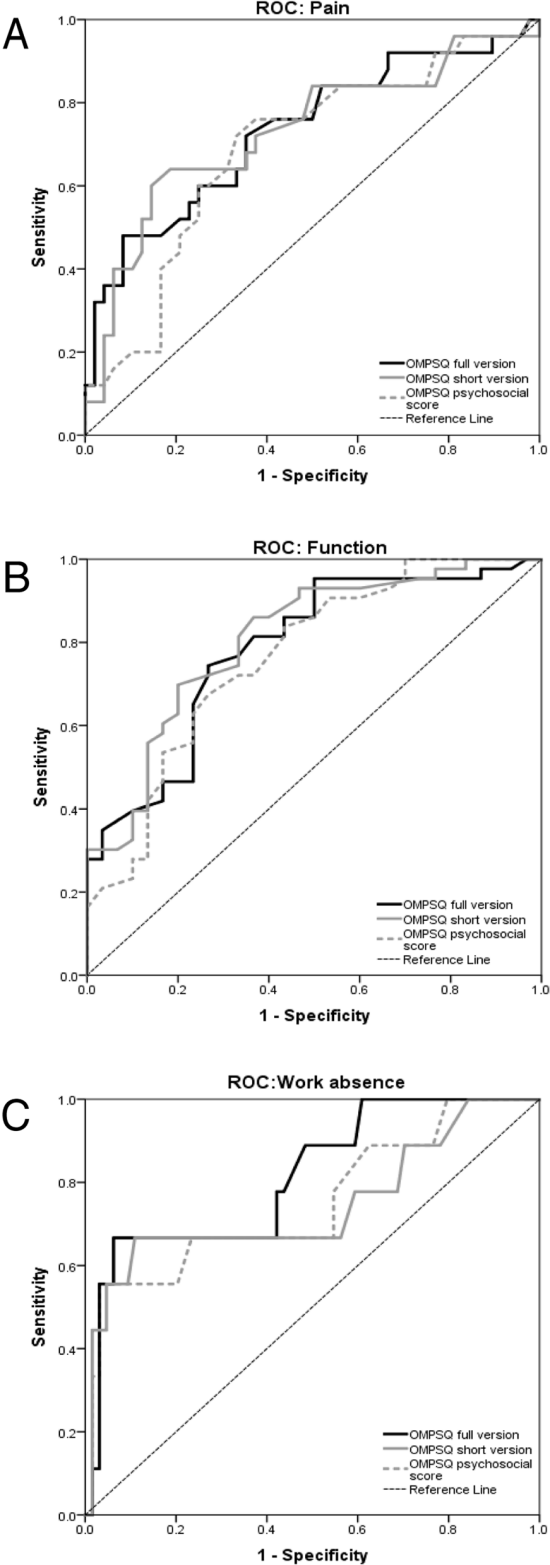
Receiver operating characteristics (ROC). Examples of ROC curves comparing full (black color), short (grey color) and psychosocial subscore (grey color, dashed line) versions of the Örebro Musculoskeletal Pain Screening Questionnaire for three outcomes: pain (**A**), function (**B**) and work absence (**C**) in the secondary care cohort

**Fig. 3:**
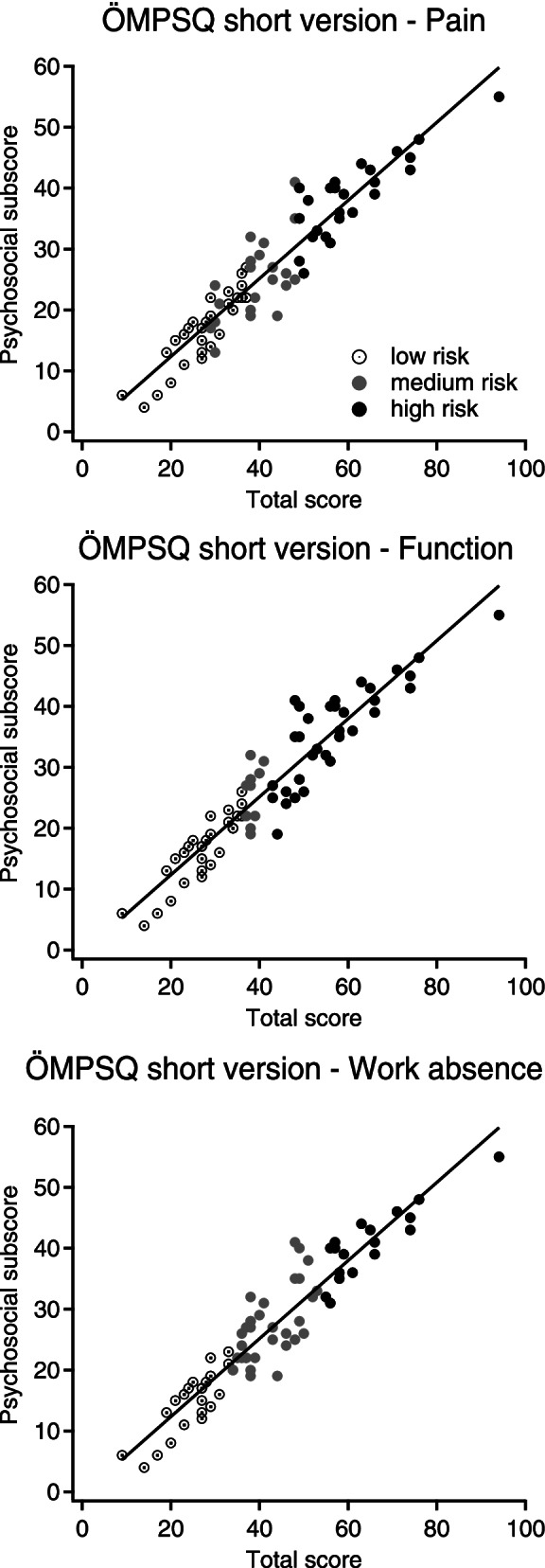
Comparison of psychosocial and total scores of the short Örebro Musculoskeletal Pain Screening Questionnaire (ÖMPSQ) version. Linear correlations between psychosocial subscore and total score of the short ÖMPSQ version are shown for low- (open circles), medium- (filled grey circles) and high- (filled black circles) risk patients taking into account three outcomes: pain (upper panel), function (middle panel) and work absence (lower panel)

In the secondary care setting (Table 3, panel A), the full, short versions and psychosocial subscore showed similar predictive values, with better discrimination for work absenteeism and function than for pain (Fig. 2). Items #7, #8 and their sum showed better predictive values for pain and function than for work absenteeism, but these differences were not statistically significant (Additional files 1 and 2). The psychosocial subscore was strongly correlated to the total score of the 10-items ÖMPSQ version (r^2^=0.876, linear regression fit) for all three outcomes (Fig. 3).

**Table 3:** Area under the ROC curve (and 95% confidence interval) measuring the accuracy of various ÖMPSQ versions and items: **good = AUC 0.8-0.89** / **fair = AUC 0.7-0.79** / poor = AUC 0.6-0.69 / *fail = AUC 0.5-0.59*

ÖMPSQ Items	Full version total score	Short version total score	Psychosocial subscore (items #5-10)	Item #7	Item #8	Item #7+#8
Outcome Variables						
A. Secondary care cohort (*n*=73)						
ÖMPSQ Pain index	**0.732** (0.605 - 0.859)	**0.730** (0.600 - 0.860)	0.690 (0.561 - 0.820)	**0.721** (0.597 - 0.845)	**0.713** (0.581 - 0.846)	**0.754** (0.636 - 0.872)
ÖMPSQ Function index	**0.784** (0.678 - 0.891)	**0.808** (0.707 - 0.909)	**0.764** (0.652 - 0.876)	**0.755** (0.642 - 0.867)	0.628 (0.501 - 0.754)	**0.786** (0.679 - 0.893)
ÖMPSQ Work Absence	**0.812** (0.658 - 0.967)	**0.745** (0.532 - 0.957)	**0.751** (0.556 - 0.946)	0.681 (0.483 - 0.879)	0.616 (0.387 - 0.845)	0.695 (0.486 - 0.905)
B. Primary care cohort, spinal pain only (*n*=91)						
Presence/absence of pain	-	0.665 (0.663-0.668)	0.633 (0.631-0.636)	0.607 (0.605-0.610)	*0.575* *(0.573-0.577)*	0.615 (0.613-0.617)
Recovery score	-	**0.822** (0.820-0.824)	**0.718** (0.715-0.721)	0.676 (0.673-0/679)	0.647 (0.644-0.649)	**0.735** (0.732-0.737)
EQ 5D pain/discomfort	-	0.654 (0.651-0.656)	0.628 (0.626-0.631)	*0.558* *(0.556-0.559)*	0.662 (0.660-0.664)	0.620 (0.618-0.623)
EQ 5D mobility	-	**0.784** (0.781-0.788)	**0.804** (0.801-0.806)	*0.575* *(0.573-0.577)*	**0.860** (0.858-0.836)	**0.770** (0.766-0.775)
EQ 5D usual activities	-	**0.745** (0.741-0.749)	**0.809** (0.807-0.811)	0.614 (0.611-0.617)	0.616 (0.612-0.620)	0.689 (0.685-0.693)
EQ 5D anxiety/depression	-	0.686 (0.683-0.688)	0.651 (0.649-0.654)	*0.560* *(0.559-0.562)*	*0.577* *(0.574-0.579)*	*0.572* *(0.570-0.575)*
C. Primary care cohort, (sub) acute pain at t0 (*n*=112)						
Presence/absence of pain	-	0.674 (0.671-0.676)	0.639 (0.637-0.641)	*0.590* *(0.588-0.592)*	*0.563* *(0.561-0.568)*	*0.589* *(0.587-0.591)*
Recovery score	-	0.666 (0.663-0.669)	0.656 (0.653-0.659)	0.625 (0.622-0.628)	*0.568* *(0.566-0.571)*	0.604 (0.602-0.607)
EQ 5D pain/discomfort	-	*0.583* *(0.581-0.586)*	0.605 (0.602-0.607)	*0.561* *(0.559-0.563)*	*0.539* *(0.537-0.541)*	*0.555* *(0.553-0.557)*
EQ 5D mobility	-	**0.750** (0.747-0.753)	**0.762** (0.759-0.764)	0.642 (0.639-0.645)	0.685 (0.682-0.688)	0.684 (0.680-0.688)
EQ 5D usual activities	-	**0.782** (0.777-0.786)	**0.837** (0.834-0.840)	0.657 (0.652-0.660)	**0.766** (0.762-0.771)	**0.721** (0.717-0.726)
EQ 5D anxiety/depression	-	0.693 (0.690-0.695)	0.683 (0.681-0.686)	*0.587* *(0.584-0.589)*	*0.587* *(0.584-0.589)*	0.611 (0.609-0.614)
D. Primary care cohort, chronic pain at t0 (*n*=292)						
Presence/absence of pain	-	**0.732** (0.730-0.733)	**0.700** (0.698-0.701)	0.646 (0.644-0.648)	0.660 (0/659-0.662)	0.695 (0.694-0.697)
Recovery score	-	**0.774** (0.773-0.776)	**0.731** (0.730-0.732)	0.629 (0.628-0.631)	0.638 (0.637-0.639)	0.683 (0.681-0.684)
EQ 5D pain/discomfort	-	**0.719** (0.718-0/720)	**0.723** (0.721-0.724)	0.638 (0.636-0.639)	0.651 (0.650-0.652)	0.689 (0.688-0.690)
EQ 5D mobility	-	**0.781** (0.779-0.782)	**0.777** (0.775-0.778)	0.644 (0.643-0.646)	**0.818** (0.817-0.819)	**0.828** (0.827-0.829)
EQ 5D usual activities	-	**0.718** (0.717-0.720)	**0.767** (0.765-0.768)	*0.580* *(0.578-0.582)*	0.699 (0.697-0.701)	**0.710** (0.708-0.712)
EQ 5D anxiety/depression	-	**0.738** (0.737-0.739)	**0.744** (0.743-0.745)	0.612 (0.610-0.613)	0.616 (0.614-0.617)	0.656 (0.655-0.658)
E. Primary care cohort, (sub) acute and chronic pain at t0, limb pain only (*n*=74)						
Presence/absence of pain	-	**0.739** (0.736-0.741)	0.611 (0.608-0.614)	0.632 (0.629-0.635)	0.691 (0.688-0.693)	0.667 (0.664-0.669)
Recovery score	-	**0.769** (0.766-0.772)	**0.705** (0.702-0.708)	**0.718** (0.715-0.721)	*0.575* *(0.572-0.579)*	0.670 (0.667-0.673)
EQ 5D pain/discomfort	-	**0.749** (0.742-0.747)	**0.711** (0.708-0.713)	0.616 (0.614-0.619)	0.649 (0.647-0.652)	0.640 (0.638-0.643)
EQ 5D mobility	-	**0.773** (0.770-0.776)	0.665 (0.662-0.669)	0.616 (0.613-0.619)	**0.775** (0.772-0.778)	**0.728** (°.725-0.731)
EQ 5D usual activities	-	0.691 (0.685-0.697)	**0.723** (0.716-0.730)	*0.489* *(not reliable)*	0.615 (not reliable)	**0.791** (0.789-0.793)
EQ 5D anxiety/depression	-	0.686 (0.682-0.690)	0.674 (0.671-0.678)	0.604 (0.601-0.607)	*0.582* *(0.579-0.586)*	*0.598* *(0.595-0.601)*
F. Primary care cohort, (sub) acute and chronic pain at t0, “non-musculoskeletal” pain only (n=24)						
Presence/absence of pain	-	0.602 (0.599-0.607)	*0.588* *(0.584-0.593)*	**0.840** (0.837-0.843)	*0.577* *(0.573-0.580)*	**0.849** (0.847-0.852)
Recovery score	-	0.671 (0.666-0.676)	0.671 (0.665-0.676)	**0.734** (0.730-0.739)	*0.598* *(0/594-0.602)*	**0.726** (0.721-0.731)
EQ 5D pain/discomfort	-	0.628 (0.624-0.632)	0.608 (0.604-0.612)	0.621 (0.618-0.624)	*0.597* *(0.594-0.610)*	0.624 (0.620-0.629)
EQ 5D mobility	-	0.671 (0.665-0.676)	**0.724** (0.719-0.729)	0.661 (0.656-0.666)	0.618 (0.613-0.622)	0.640 (0.635-0.645)
EQ 5D usual activities	-	**0.814** (0.810-0.817)	**0.783** (0.779-0.787)	0.612 (0.608-0.616)	**0.915** (0.912-0.917)	0.686 (0.681-0.692)
EQ 5D anxiety/depression	-	**0.923** (0.921-0.927)	**0.915** (0.912-0.918)	**0.704** (0.699-0.709)	**0.828** (0.823-0.833)	**0.808** (0.804-0.813)
G. Primary care cohort, all patients (n=542)						
Presence/absence of pain	-	**0.742** (0.741-0.743)	0.692 (0.691-0.693)	0.687 (0.686-0.688)	0.642 (0.640-0.643)	**0.700** (0.699-0.702)
Recovery score	-	**0.773** (0.772-0.774)	**0.719** (0.718-0.721)	0.662 (0.661-0.663)	0.637 (0.636-0.638)	0.692 (0.691-0.693)
EQ 5D pain/discomfort	-	**0.726** (0.725-0.727)	**0.709** (0.708-0.710)	0.680 (0.679-0.681)	0.638 (0.637-0/639)	**0.701** (0.700-0.702)
EQ 5D mobility	-	**0.782** (0.781-0.783)	**0.780** (0.779-0.782)	0.668 (0.667-0.669)	**0.800** (0.799-0.802)	**0.807** (0.806-0.808)
EQ 5D usual activities	-	**0.752** (0.751-0.754)	**0.790** (0.788-0.791)	0.624 (0.623-0.627)	**0.718** (0.716-0.719)	**0.738** (0.737-0.740)
EQ 5D anxiety/depression	-	**0.736** (0.735-0.737)	**0.733** (0.731-0.734)	0.622 (0.621-0.623)	0.618 (0.617-0.619)	0.661 (0.660-0.662)

***ÖMPSQ*** Örebro Musculoskeletal Pain Screening Questionnaire, ***ROC*** Receiver Operating Characteristic, ***AUC*** Area under the curve.

Not reliable: when results of bootstrapping for CI are very different from initial AUC calculation (difference leading to change of result category) & visual control of the ROC curve confirms a bad fit

Data from the primary care patients (Table 3, panel B) were comparable to those of our first cohort: the short ÖMPSQ questionnaire and its psychosocial subscore had a fair to good predictive value for the recovery index as well as functional parameters (EQ-5D-5L mobility and EQ-5D-5L usual activities). Prediction of pain persistence or mood disturbance was poor. Items #7, #8 and their sum also mostly had poor predictive ability.

### Predictive value of the French short ÖMPSQ in patients with (sub) acute and chronic pain (Table 3, panels C-D)

For patients with (sub) acute pain, the short ÖMPSQ total and psychosocial subscores showed poor predictive abilities for pain outcomes (presence of pain, the composite recovery score and pain assessed by EQ-5D-5L) as well as anxiety/depression (EQ-5D-5L), and fair to good validity for functional outcomes (EQ-5D-5L mobility and ability to do usual activities). Items #7, #8 and their sum only showed poor to good predictive abilities for functional outcomes.

In patients with chronic pain, the short ÖMPSQ and its subsets showed better predictive abilities than in patients with (sub) acute pain: fair results were observed for all outcomes for the total ÖMPSQ and the psychosocial subscore. Items #7, #8 and their sum usually showed poor validity, except for prediction of mobility, which was fair to good.

### Predictive value of the French short ÖMPSQ in patients with pain of different localizations (Table 3, panels B, E, F)

As said in Methods, for this analysis, data from patients presenting pain in more than one site were excluded. Both in patients with only spinal or limb pain, the short ÖMPSQ total score and the psychosocial subscore showed poor to good results depending on the outcome variable. Items #7, #8 and their sum gave heterogeneous results, mostly of poor quality.

Data concerning patients with only presumed “non-MSKP” should be interpreted with caution because of the small number of observations (n = 24). Results were heterogeneous, the ÖMPSQ and its subsets had a poor to good predictive validity, depending on the variable considered.

### Cutoff scores (Table 4)

For the short ÖMPSQ version, we propose two cutoff scores for each of the three outcomes, with either good sensitivity (80%) or good specificity (80%) to distinguish three groups of patients with low, intermediate, and high risk of chronicity.

**Table 4:** Proposed cut-off scores (Low cut-off: Sensitivity 80%, High cut-off: Specificity 80%) determining three risk groups using short forms of ÖMPSQ. Data are formatted according to the results of the ROC AUC analysis: **good =AUC O8-0.89** / **fair = AUC 0.7-0.79** / poor = AUC 0.6-0.69 / *fail = AUC 0.5-0.59*

Outcome variables	ÖMPSQ short version Total score		ÖMPSQ psychosocial subscore cut-off scores (Items #5-10)		ÖMPSQ cut-off scores for Items #7+#8	
	Low cut-off	High cut-off	Low cut-off	High cut-off	Low cut-off	High cut-off
A. Secondary care cohort (*n*=73)						
ÖMPSQ Pain index	**37**	**48**	22	39	**5**	**10**
ÖMPSQ Function index	**36**	**42**	**22**	**28**	**4**	**8**
ÖMPSQ Work absence	**33**	**53**	**22**	**36**	4	12
B. Primary care cohort, patients with spinal pain (*n*=91)						
Presence/absence of pain	38	51	20	32	5	9
Recovery score	**46**	**51**	**23**	**31**	**7**	**9**
EQ 5D pain/discomfort	39	51	20	31	4	9
EQ 5D mobility	**47**	**53**	**29**	**33**	**10**	**10**
EQ 5D usual activities	**40**	**52**	**29**	**32**	7	10
EQ 5D anxiety/depression	41	52	20	32	*4*	*10*
C. Primary care cohort, (sub) acute pain at t0 (*n*=112)						
Presence/absence of pain	36	47	19	20	*2*	*8*
Recovery score	36	49	21	31	*2*	*8*
EQ 5D pain/discomfort	34	49	20	30	2	8
EQ 5D mobility	**37**	**48**	**23**	**30**	2	8
EQ 5D usual activities	**37**	**49**	**29**	**30**	**2**	**8**
EQ 5D anxiety/depression	38	48	20	30	2	8
D. Primary care cohort, chronic pain at t0 (*n*=292)						
Presence/absence of pain	**41**	**50**	**20**	**28**	6	10
Recovery score	**48**	**54**	**24**	**33**	8	13
EQ 5D pain/discomfort	**44**	**55**	**22**	**31**	7	11
EQ 5D mobility	**52**	**58**	**29**	**36**	**11**	**12**
EQ 5D usual activities	**47**	**57**	**29**	**35**	**9**	**12**
EQ 5D anxiety/depression	**49**	**57**	**26**	**35**	8	14
E. Primary care cohort, (sub) acute and chronic pain at t0, limb pain only (*n*=74)						
Presence/absence of pain	**36**	**43**	13	26	3	9
Recovery score	**38**	**50**	**16**	**30**	5	11
EQ 5D pain/discomfort	**39**	**47**	**16**	**26**	4	11
EQ 5D mobility	**41**	**46**	15	27	**6**	**10**
EQ 5D usual activities	37	48	**18**	**27**	**11**	**11**
EQ 5D anxiety/depression	40	49	16	29	*4*	*11*
F. Primary care cohort, (sub) acute and chronic pain at t0, “non-musculoskeletal” pain only (*n*=24)						
Presence/absence of pain	35	49	*18*	*30*	**5**	**6**
Recovery score	49	55	22	40	**7**	**10**
EQ 5D pain/discomfort	33	50	19	31	5	9
EQ 5D mobility	36	55	22	31	aberrant values	
EQ 5D usual activities	**51**	**51**	**30**	**34**	6	10
EQ 5D anxiety/depression	**49**	**50**	**26**	**30**	**6**	**9**
G. Primary care cohort, all patients (*n*=542)						
Presence/absence of pain	**39**	**49**	19	28	**5**	**10**
Recovery score	**47**	**53**	**22**	**32**	7	11
EQ 5D pain/discomfort	**41**	**52**	**21**	**31**	**5**	**10**
EQ 5D mobility	**49**	**55**	**28**	**33**	**10**	**11**
EQ 5D usual activities	**45**	**55**	**28**	**33**	**8**	**11**
EQ 5D anxiety/depression	**44**	**55**	**25**	**33**	6	12

*ÖMPSQ* Örebro Musculoskeletal Pain Screening Questionnaire

Data showed a large variability, depending on the population and the outcome variable chosen. Cutoff scores calculated for the secondary care population are lower than those observed for the primary care patients. Cutoff scores for patients with chronic pain tended to be higher than those for patients with (sub) acute pain. Cutoffs from the total short ÖMPSQ score showed only small differences when comparing patients with spinal pain *versus* limb pain, both of which being probably mainly musculoskeletal. When expressed relatively to the maximal score of each subset, cutoff variability was much larger for items #7+#8 than for the psychosocial subscore, followed by the total short ÖMPSQ score (27, 15 and 10% respectively). This suggests that cutoff determination is more sensitive to population differences when only a small number of risk factors is considered, as in the ÖMPSQ subsets.

## Discussion

Secondary and tertiary prevention of chronic pain require accurate identification of modifiable risk factors. In busy clinical settings, there is a need for simple tools allowing for the rapid detection of these factors. The ideal tool would be a short questionnaire, with generic cutoff scores, that could be used in a large range of disorders and clinical settings. In this perspective, the short ÖMPSQ, designed to evaluate patients with MSKP, but assessing most of the risk factors also identified in non-MSKP disorders, deserved further exploration.

Our data show that the short French ÖMPSQ and its subsets had predictive properties comparable to those of the full French ÖMPSQ version in patients suffering from spinal pain in (sub) acute patients consulting in a secondary care setting. These properties were globally confirmed in a more heterogeneous population of patients consulting in a primary care setting, suffering from pain of variable duration (acute, (sub) acute, and chronic) and localization (spinal, limb, “probably non-MSK”). However, increasing population heterogeneity resulted in slightly worse predictive performance and more variable cutoff scores than in our first cohort. The short ÖMPSQ showed fair to good predictive performance for chronic pain patients. Data obtained for spinal and limb pain suggest that the short ÖMPSQ has an acceptable predictive validity for a large range of patients suffering from MSKP.

These data are in line of those of the original validation of the short ÖMPSQ version [9] as well as other studies [11, 12] and confirm that the ÖMPSQ performs better for functional than pain-related outcomes. This, as well as the observation that cutoff scores tended to be higher for functional limitation items and work absence than for pain outcomes, suggest that a significant proportion of patients remain reasonably functional despite persistent pain.

The choice of cutoff scores, which will influence patients’ treatment assignment, is an important but difficult process. In their original study, Linton et al. [9] proposed a cutoff score of 50 (out of 100) for the 10-item ÖMPSQ, to distribute patients into two risk groups, but other authors suggested that it might be more accurate to distinguish three risk groups [10, 13, 16]. For this reason, we proposed two cutoff scores for each of the three outcomes, to identify low-, medium- and high-risk groups, with either good sensitivity (80%) or good specificity (80%). However, the choice of cutoff points could be influenced by dichotomization in ROC analysis, choice of threshold scores and time points of administering the questionnaire after the onset of pain. Moreover, we observed that increasing population heterogeneity increased the variability of cutoff scores. These observations illustrate the limitation of prognostic tools, which are very sensitive to subjective choices as well as patient populations and indicate that it might be difficult to choose universal cutoff scores. Other criteria, such as the available resources for patient management, should be considered and the results of tools such as the ÖMPSQ must be integrated into the whole patient context.

An alternative to the use of rigid cutoff scores is to analyze patients’ answers to specific questions, alone or in combination with the total short ÖMPSQ score. The fair performances of single questions about patient’s expectations confirmed that the patient’s own beliefs are significant predictors of recovery [10, 46–48]. Taking these beliefs into account and using a psychosocial subscore might have an impact when developing targeted treatment. For patients with acute low-back pain, stratifying care according to the SBT improved patient disability outcomes and halved time off work, without increasing healthcare cost [3, 49]. One could use a similar approach for treatment recommendations for risk groups obtained using the ÖMPSQ, but this approach needs validation.

The fair to good predictive performance of the short ÖMPSQ for chronic pain patients confirms that chronic pain is not a fixed state [33] and stresses the potential interest of tertiary prevention strategies. Our study also observed fair predictive performances of the short ÖMPSQ for patients suffering from limb pain, confirming that the risk factors in this population are probably mostly similar to those of spinal pain patients. The available data were not sufficient to conclude on the validity of the short ÖMPSQ for patients with “probably non-MSKP”, but the results are encouraging. The low proportion of patients with “probably non-MSKP” in our study is probably attributable to both exclusion of patients with more than one pain site for the subgroup analysis and the relatively low proportion of these pain localization, when compared to MSKP [21].

To our knowledge this is the first study that showed the interest of psychosocial subscore of the ÖMPSQ, in line with the observations made with the SBT. This observation confirmed therefore the importance of addressing psychosocial factors in the management of at-risk patients. However, the clinical efficiency of treatment assignment based on this psychosocial subscore remains to be tested and the moderate predicting value of this subscore indicates that other factors play a role in chronicity. Another important finding was the validity of the short ÖMPSQ to predict the evolution of patients with limb pain. Finally, we confirmed the validity of the short ÖMPSQ for patients with chronic pain, thus fostering a prognostic definition of chronic pain [33].

This study has some limitations. (1) Participants in the secondary care cohort completed the full and not the short ÖMPSQ version. The impact of this is probably small, according to previous suggestions [9] and to the results from the primary care patients. (2) Participant’s attrition was high, as often in longitudinal studies, and did not allow excluding a selection bias, limiting the external validity of our results. (3) In the secondary care cohort, all three outcomes were derived from the ÖMPSQ itself, with the risk of circular reasoning. However, our previous work showed similar results when using the Oswestry disability index to assess functional outcomes [13], and in primary care patients, an independent tool (EQ-5D-5L) was used to evaluate patients’ health status at one year. (4) The diagnosis of “MSKP” versus “probably non-MSKP” was based on the patients’ description of pain localization and not on a medical diagnosis. We are aware that, for example, calf pain may have an arteritic cause while thoracic or abdominal pain may have a parietal MSK origin. However, for practical reasons, it would not have been possible nor reliable to ask the general practitioners to record a diagnosis, given the heterogeneity of the clinical situations, the need to interfere as little as possible with the clinical consultation (to reduce the burden of the study and increase its acceptability for the general practitioners) and the difficulty of providing an “instant diagnosis” in some instances where further investigation (i.e., imaging) might be necessary. Therefore, the distinction between “MSK” and “non-MSK” pain must be considered with caution.

## Conclusions

The French version of the short ÖMPSQ and its subsets are useful tools for screening patients suffering from acute, subacute, and chronic spinal and limb pain. However, the choice of cutoff scores might be difficult and should integrate the clinical objectives (i.e., reducing long-term pain and/or function and/or work absenteeism) as well as the available therapeutic resources. We would advise clinicians not to stick to a single score but use this questionnaire as a guide to open a discussion with the patient, allowing a more detailed and enlarged assessment of his beliefs, behaviors and resources. The management decisions should integrate the results from this questionnaire to the context of the patient, his own objectives and the available resources

## Supplementary Information

**Additional file 1. **Comparison of area under the receiver operating characteristic curves for various Örebro Musculoskeletal Pain Screening Questionnaire versions for three outcomes (work absence, function and pain) in the secondary care cohort. **Additional file 2.** Comparison of area under the receiver operating characteristic curves between three outcomes (work absence, function and pain) for different Örebro Musculoskeletal Pain Screening Questionnaire versions in the secondary care cohort.

## Data Availability

The datasets used and/or analyzed during the current study are available from the corresponding author upon reasonable request.

## Abbreviations

ÖMPSQ: Örebro Musculoskeletal Pain Screening Questionnaire
LBP: Low Back Pain
SBT: STarT Back Screening Tool
RTW: Return To Work
MSKP: Musculoskeletal Pain
ROC: Receiver Operating Characteristic
AUC: Area Under the ROC Curve
IASP: International Association for the Study of Pain
