# Supplementary material for: Can the French version of the short Örebro Musculoskeletal Pain Screening Questionnaire or its subsets predict the evolution of patients with acute, (sub) acute and chronic pain?
Source: BMC Musculoskelet Disord. 2022 Apr 1;23:311. doi: 10.1186/s12891-021-04944-9 (PMC8976369; doi:10.1186/s12891-021-04944-9)
Supplement: Supplementary file 1 — Additional file 1. Comparison of area under the receiver operating characteristic curves for various Örebro Musculoskeletal Pain Screening Questionnaire versions for three outcomes (work absence, function and pain) in the secondary care cohort. Additional file 2. Comparison of area under the receiver operating characteristic curves between three outcomes (work absence, function and pain) for different Örebro Musculoskeletal Pain Screening Questionnaire versions in the secondary care cohort. [file 12891_2021_4944_MOESM1_ESM.docx]

**Additional file 1.**

**Table 1**. Comparison of ROC AUC for various ÖMPSQ versions for three outcomes (work absence, function and pain) in the secondary care cohort.

| **ÖMPSQ**  **version**  **Outcome**  **variable** | **Work absence**  **(AUC ± SD)**  Negative cases = 64  Positive cases = 9 | **Function index**  **(AUC ± SD)**  Negative cases = 30  Positive cases = 43 | **Pain index**  **(AUC ± SD)**  Negative cases = 48  Positive cases = 25 |
| --- | --- | --- | --- |
| **ÖMPSQ full**  **OMPSQ short** | 0.812 ± 0.079  0.745 ± 0.108  (p =0.615) | 0.784 ± 0.055  0.808 ± 0.052  (p =0.741) | 0.732 ± 0.065  0.730 ± 0.067  (p =0.983) |
| **ÖMPSQ short**  **ÖMPSQ psychosocial**  **(items 5-10)** | 0.745 ± 0.108  0.751 ± 0.100  (p =0.965) | 0.808 ± 0.052  0.764 ± 0.057  (p =0.553) | 0.730 ± 0.067  0.690 ± 0.066  (p =0.671) |
| **ÖMPSQ psychosocial**  **(items 5-10)**  **ÖMPSQ item 7** | 0.751 ± 0.100  0.681 ± 0.101  (p =0.623) | 0.764 ± 0.057  0.755 ± 0.057  (p =0.908) | 0.690 ± 0.066  0.721 ± 0.063  (p =0.743) |
| **ÖMPSQ** **item 7**  **ÖMPSQ** **item 8** | 0.681 ± 0.101  0.616 ± 0.117  (p =0.660) | 0.755 ± 0.057  0.628 ± 0.065  (p =0.139) | 0.721 ± 0.063  0.713 ± 0.067  (p =0.932) |
| **ÖMPSQ** **item 8**  **ÖMPSQ** **item 8+7** | 0.616 ± 0.117  0.695 ± 0.107  (p =0.592) | 0.628 ± 0.065  0.786 ± 0.055  (p =0.06) | 0.713 ± 0.067  0.754 ± 0.060  (p =0.655) |

**ÖMPSQ** – Örebro Musculoskeletal Pain Screening Questionnaire; **ROC** – Receiver Operating Characteristic; **AUC** – Area Under the Curve; **SD** – Standard Deviation; **p-value** - Chi-square test (two tailed).[1, 2]

**Additional file 2**

**Table 2**. Comparison of ROC AUC between three outcomes (work absence, function and pain) for each ÖMPSQ version in the secondary care cohort.

| **ÖMPSQ full version** | Pain  Function | 0.732 ± 0.065  0.784 ± 0.055 | p = 0.543 |
| --- | --- | --- | --- |
|  | Function  Work | 0.784 ± 0.055  0.812 ± 0.079 | p = 0.788 |
|  | Pain  Work | 0.732 ± 0.065  0.812 ± 0.079 | p = 0.471 |
| **ÖMPSQ short version** | Pain  Function | 0.730 ± 0.067  0.808 ± 0.052 | p = 0.342 |
|  | Function  Work | 0.808 ± 0.052  0.745 ± 0.108 | p = 0.568 |
|  | Pain  Work | 0.730 ± 0.067  0.745 ± 0.108 | p = 0.899 |
| **ÖMPSQ psychosocial score items 5-10** | Pain  Function | 0.690 ± 0.066  0.764 ± 0.057 | p = 0.397 |
|  | Function  Work | 0.764 ± 0.057  0.751 ± 0.100 | p = 0.907 |
|  | Pain  Work | 0.690 ± 0.066  0.751 ± 0.100 | p = 0.609 |
| **ÖMPSQ short item 7** | Pain  Function | 0.721 ± 0.063  0.755 ± 0.057 | p = 0.694 |
|  | Function  Work | 0.755 ± 0.057  0.681 ± 0.101 | p = 0.529 |
|  | Pain  Work | 0.721 ± 0.063  0.681 ± 0.101 | p = 0.744 |
| **ÖMPSQ short item 8** | Pain  Function | 0.713 ± 0.067  0.628 ± 0.065 | p = 0.362 |
|  | Function  Work | 0.628 ± 0.065  0.616 ± 0.117 | p = 0.923 |
|  | Pain  Work | 0.713 ± 0.067  0.616 ± 0.117 | p = 0.437 |
| **ÖMPSQ short item 7+8** | Pain  Function | 0.754 ± 0.060  0.786 ± 0.055 | p = 0.697 |
|  | Function  Work | 0.786 ± 0.055  0.695 ± 0.107 | p = 0.430 |
|  | Pain  Work | 0.754 ± 0.060  0.695 ± 0.107 | p = 0.625 |

**ÖMPSQ** – Örebro Musculoskeletal Pain Screening Questionnaire; **ROC** – Receiver Operating Characteristic; **AUC** – Area Under the Curve; **SD** – Standard Deviation; **p-value** - Chi-square test (two tailed).[1, 2]

Reference List

[1]. DeLong ER, DeLong DM and Clarke-Pearson DL. Comparing the areas under two or more correlated receiver operating characteristic curves: a nonparametric approach. Biometrics. 1988;44(3):837-45.

[2]. Hanley JA and McNeil BJ. A method of comparing the areas under receiver operating characteristic curves derived from the same cases. Radiology. 1983;148(3):839-43.
